# Atypical progression of delayed encephalopathy post-carbon monoxide poisoning with fluctuating psychotic symptoms: a case report

**DOI:** 10.3389/fpsyt.2025.1633732

**Published:** 2025-08-01

**Authors:** Yunhan Lin, Yan Shao, Xinyu Sun

**Affiliations:** ^1^ Peking University Sixth Hospital, Beijing, China; ^2^ National Clinical Research Center for Mental Disorders, Beijing, China

**Keywords:** delayed encephalopathy after acute carbon monoxide poisoning, DEACMP, neuropsychiatric sequelae, carbon monoxide poisoning, cognitive function, case report

## Abstract

**Objectives:**

This report details a case of delayed encephalopathy after acute carbon monoxide poisoning (DEACMP), a condition with significant neuropsychiatric sequelae that is often underrecognized. The case is notable for its atypical progression and poor response to conventional treatments, highlighting the need for awareness and novel approaches in managing similar cases.

**Case report:**

Mrs. C, a 53-year-old female with a long history of recurrent depression, suffered from severe carbon monoxide poisoning. Despite initial recovery, she exhibited a relapse marked by profound cognitive decline and erratic behaviors such as inappropriate urination and fecal smearing. The diagnostic workup, including MRI and neuropsychological testing, confirmed DEACMP. Various treatments were employed with limited success. Her course of illness underscores the fluctuating nature of her symptoms and the overall decline in her cognitive function.

**Conclusions:**

This case underscores the complex clinical management and refractory nature of DEACMP, emphasizing the necessity for comprehensive, individualized treatment approaches. The insights provided here advocate for heightened surveillance during the ‘false recovery period’ and tailored therapeutic strategies to alleviate symptoms, improve quality of life, and minimize long-term neurological damage in patients suffering from DEACMP.

## Introduction

Globally, carbon monoxide (CO) poisoning is a common acute toxicological emergency, posing a significant public health challenge (1). Epidemiological data reveal that in United States alone, there are over 50,000 emergency incidents related to carbon monoxide poisoning each year (2), with comparable occurrences noted in China (3). High incidence rates are particularly common in environments where heating appliances and indoor combustion devices are improperly utilized. The primary etiology is the markedly higher binding affinity of CO for hemoglobin compared to oxygen, leading to a spectrum of clinical manifestations that affect multiple organ systems (4, 5). The nervous system is particularly vulnerable, as CO exposure can lead to a substantial increase in neuronal damage, manifesting in severe neurological effects (6). Acute exposure may result in non-specific symptoms such as headaches, nausea, and drowsiness in mild cases, escalating to unconsciousness, comatose states, or even mortality in severe instances. Remarkably, days to months after recovery from acute symptoms, typically between 2 to 60 days, a period also known as the “false recovery period”, new neuropsychiatric symptoms can emerge, including cognitive impairment, memory degradation, alterations in behavior and personality, as well as motor dysfunction. This condition, named DEACMP, has been reported widely (7, 8), with the prevalence of post-CO poisoning encephalopathy exhibiting considerable variability among those affected, ranging from 13% to 50% (9). This case presents many characteristics common to carbon monoxide-induced delayed encephalopathy but also features unique aspects. The distinctive attributes of this case are delineated in the subsequent sections.

## Case report

Mrs. C is a 53-year-old married woman with a primary school education, working in retail. She has a history of depression for over 20 years and suffered from encephalopathy due to carbon monoxide (CO) poisoning for more than a year. She has experienced depressive symptoms for the past six months, along with recurrent behavioral abnormalities and cognitive decline for over two months.

Over 20 years ago, Mrs. C first exhibited signs of depression, including low mood, reduced interest, lack of energy, insomnia, and lack of appetite. She responded well to antidepressant treatment, with complete improvement after each course of medication. However, during the 20 years of continuous medication, she experienced several episodes of depression, each triggered by life events. After each episode, she switched antidepressant medications, including venlafaxine, duloxetine, and sertraline, although the exact dosages are unclear. After changing medications, her condition improved quickly, and the symptoms were generally the same during each recurrence.

At the end of 2022, Mrs. C and her husband used charcoal for heating at home, which led to CO poisoning while they were asleep. The total exposure time exceeded 10 hours. Mr. C did lose consciousness, while Mrs. C didn’t and her clarity of consciousness decreased, accompanied by incontinence. Both were sent to the local emergency department by their son, where a head CT scan showed bilateral basal ganglia softening lesions. Both were treated with hyperbaric oxygen(HBO) therapy (specific parameters unknown), and their symptoms quickly improved. However, about a month later, their conditions worsened. Mrs. C’s symptoms were more severe, presenting with a staggered gait, ataxia, and incomplete orientation, along with irrelevant responses, and she became unable to care for herself, needing assistance with personal hygiene and eating. After continuing HBO treatment for three months, Mrs. C gradually improved, returning to her pre-illness social function and was able to communicate normally and assist her husband with his retail business.

Six months ago, after experiencing the deaths of family members and the severe illness of a close relative, Mrs. C gradually developed depressive symptoms again. Unlike previous episodes, this time the symptoms lasted longer and her response to medication was poor. Additionally, more than two months ago, she suddenly exhibited several abnormal behaviors, such as urinating outside the toilet, smearing feces on the toilet seat, and repeatedly asking for a suppository. These behaviors fluctuated, with episodes focused entirely on defecation-related thoughts, while during the intervals, she could communicate clearly. Her family took her to the local psychiatric department, where treatment with sulpiride injections alleviated the symptoms for a week before they relapsed.

**Figure 1:**
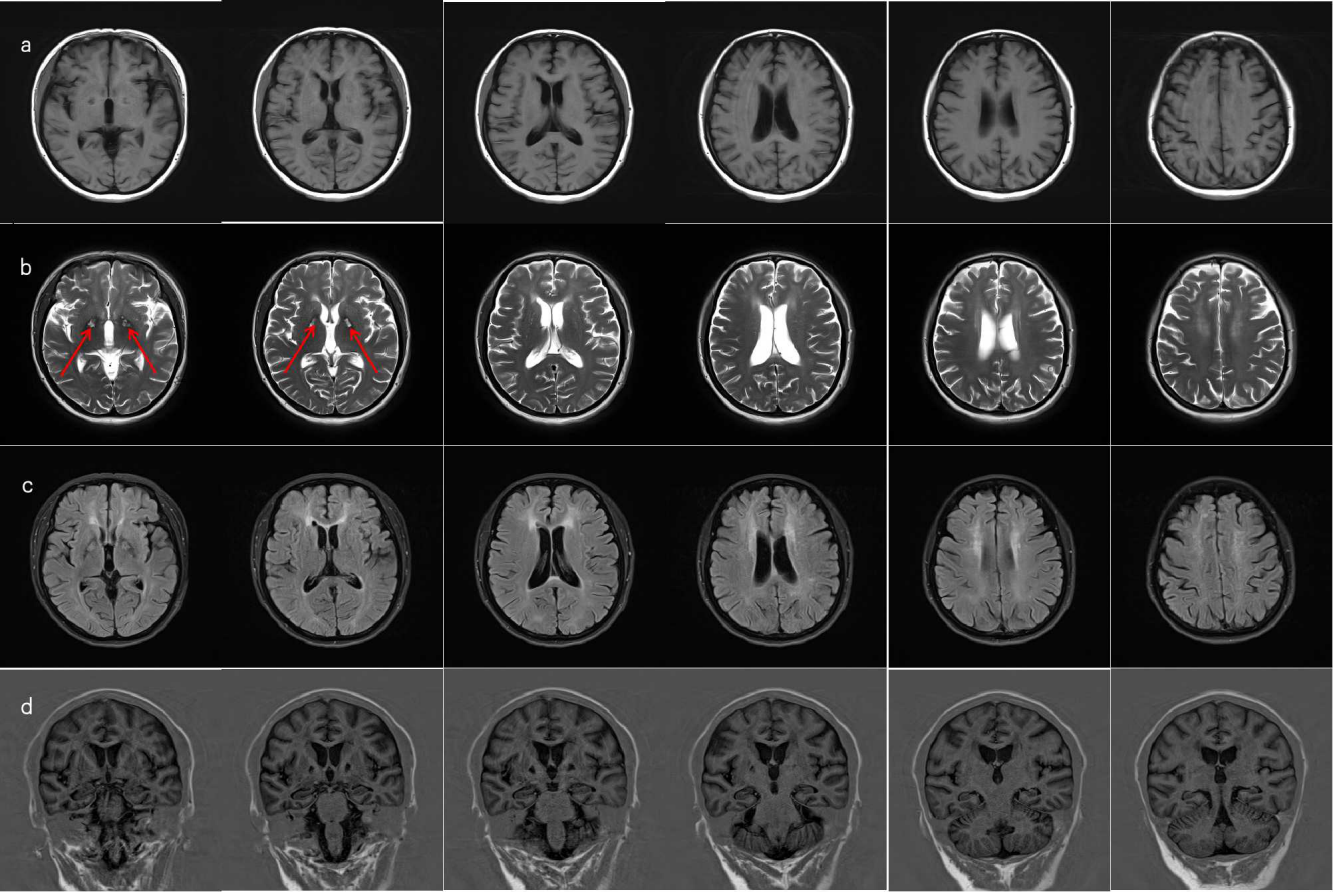
MRI of brain. **(a)** On T1-weighted imaging (T1WI), there is visible symmetric band-like slightly hypointense signal in the bilateral basal ganglia regions, with symmetric dot-like and patchy slightly hypointense signals adjacent to the bilateral lateral ventricles and in both frontal lobes. **(b)** On T2-weighted imaging (T2WI), there is visible symmetric band-like slightly hyperintense signal with a surrounding hypointense ring in the bilateral basal ganglia areas (red arrows), and symmetric dot-like and patchy slightly hyperintense signals adjacent to the bilateral lateral ventricles and in both frontal lobes. **(c)** On FLAIR imaging, there is visible symmetric band-like high signal in the bilateral basal ganglia areas, adjacent to the bilateral lateral ventricles, and in both frontal lobes. **(d)** The hippocampal angles revealed a marginally widened bilateral choroid fissure.

Upon admission, Mrs. C underwent a full set of examinations, including cognitive testing, which showed an MMSE score of 24 and a MoCA score of 16. Head MRI results (**Figure 1**) indicated abnormal signals in both basal ganglia regions (with red arrows to highlight), along with white matter demyelination and a cyst in the right maxillary sinus. Routine EEG showed preserved posterior alpha rhythm with no epileptiform or focal abnormalities; NIRS revealed task-related prefrontal haemodynamic fluctuations, and P300 event-related potentials indicated attenuated responses complicated by artefacts (detailed electrophysiological findings are provided in **Supplementary Material**). Additionally, laboratory results showed elevated blood sugar levels, leading to a diagnosis of diabetes. Other laboratory results were unremarkable.

Based on these findings, a multidisciplinary consultation was conducted. According to the ICD-10 diagnostic criteria, Mrs. C was diagnosed with a psychiatric disorder due to brain damage and functional abnormalities or physical illness. Neurologists consulted on the case and concluded that the patient’s condition was related to her previous history of CO poisoning.

Initial treatment included ginkgo biloba extract, the antipsychotic quetiapine, and the sedative clonazepam. Quetiapine was gradually titrated to 50 mg/day but later reduced due to concerns over cognitive side effects. Although sleep improved slightly, disinhibited behaviors persisted, and cognitive function continued to decline (MMSE dropped to 22, MoCA to 11). Further neuropsychological assessment using the Repeatable Battery for the Assessment of Neuropsychological Status revealed impairments in memory and verbal domains (**Table 1**). Given the progressive behavioral and emotional disturbances—particularly the patient’s disinhibited behaviors, which severely strained her family relationships and induced marked anxiety—an antidepressant (escitalopram) and mood stabilizer (magnesium valproate) were added. Donepezil was also briefly introduced for cognitive support but later replaced with memantine. Despite these pharmacological interventions, improvement remained limited. The patient underwent ten sessions of HBO in April, which yielded no clinical benefit. In May, modified electroconvulsive therapy (mECT) was initiated. After seven sessions, disinhibited behaviors showed partial improvement; however, cognitive decline worsened (MMSE dropped to 15), prompting discontinuation of mECT. Memantine was then gradually titrated up to 10 mg/day. Escitalopram and magnesium valproate dosages were further adjusted accordingly. At discharge in early June, the patient remained cognitively impaired with residual disinhibition, though agitation had partially improved. During the three-month follow-up, her condition remained unstable, characterized by alternating episodes of lucidity and confusion, as well as persistent anxiety and agitation. The dosage of escitalopram was increased, resulting in some emotional stabilization, but behavioral symptoms continued to be problematic. She remained homebound and required constant caregiving. In subsequent outpatient follow-ups (from July onward), a more activating pharmacological strategy was adopted: bupropion was added and titrated to 225 mg/day, in combination with escitalopram, magnesium valproate, and memantine. Over the following six months, the patient exhibited substantial behavioral improvement. Disinhibited behaviors nearly resolved, and her ability to perform daily tasks gradually returned. She continued to experience transient morning cognitive slowing—such as difficulty concentrating and responding appropriately during conversations—but this improved over time. By the afternoons, she could perform household chores and communicate more fluently.

**Table 1:** Cognitive Function Tests during Admission.

		Admission	Ten days after	One month after
MMSE	Orientation	9	7	4
	Immediate Memory	3	3	3
	Attention and Calculation	5	3	1
	Delayed Recall	0	2	0
	Language Skills	7	7	7
	Total Score	24	22	15
MoCA	Visuospatial and Executive Function	2	2	can’t complete
	Naming	1	1	
	Attention	5	4	
	Language	1	0	
	Abstract Thinking	1	1	
	Delayed Recall	0	0	
	Orientation	6	3	
	Total Score	16	11	
RBANS*	Immediate Memory	not performed	53	can’t complete
	Visuospatial/constructional		78	
	Language		54	
	Attention		88	
	Delayed Memory		48	

*Repeatable Battery for the Assessment of Neuropsychological Status Effort Scale (RBANS) (Standard score).

**Figure 2:**
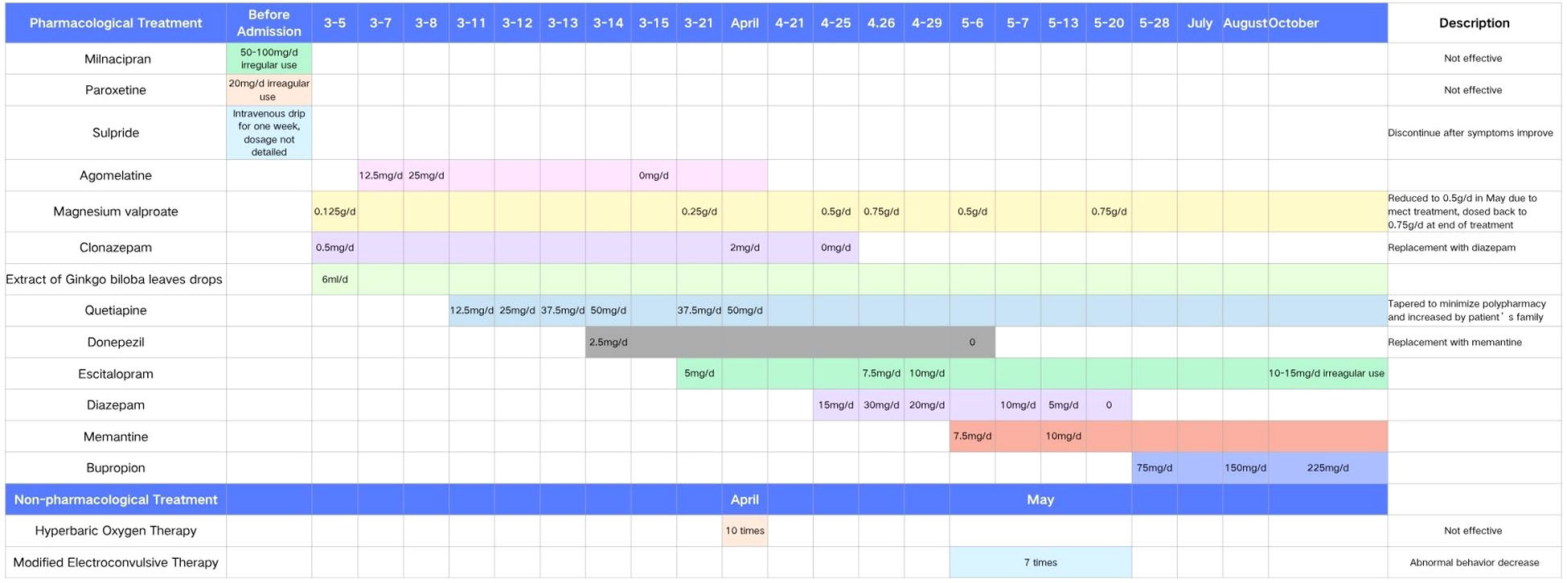
Timeline of pharmacological and non-pharmacological treatments during hospitalization and follow-up. The chart displays the initiation, discontinuation, and dose adjustments of key medications from admission in early March through October.

Changes in cognitive function over the course of treatment are shown in **Table 2**, and the detailed pharmacological timeline is summarized in **Figure 2**.

**Table 2:** Timeline of events.

Date	Event	Description
2022-12	CO poisoning and Acute treatment	Both Mr. and Mrs. C exposed to carbon monoxide at home. Immediate emergency treatment (oxygen therapy) administered.
2022-12	Initial pseudo-recovery	Returned home with normal cognitive function (both patients).
2023-01	Onset of delayed encephalopathy and treatment	Mrs. C developed cognitive decline, disinhibition, and psychosis. Mr. C showed mild transient symptoms. Maintain hyperbaric oxygen therapy.
2023-01- 2023-08	Functional improvement plateau	Mr. C recovered normal cognitive function quickly, while Mrs. C gradually functional recovery in a slowly speed and was able to resume independent daily activities and return to work
2023-08	Fourth depressive episode	Recurrence depressive symptoms and lasted longer than before.
2024-01	Disinhibition and cognitive dysfunction	Marked decline in function; reappearance of psychomotor retardation and negative symptoms.
2024-03	Psychiatric admission	Admitted to psychiatric unit due to worsening behavior. Brain MRI, neurocognitive testing. Start of combined pharmacological and non-pharmacological treatments.
2024-05	Discharged	Partial remission; continued outpatient therapy and medication.
2024-05–2024-12	Gradual cognitive recovery	Progressive improvement in daily function and mood; neurocognitive scores improved but did not return to baseline.
2024-12	Follow-up	Final review; mild executive deficits and cognitive dysfunction remain, but stable and manageable. No recurrence of psychosis or major depressive symptoms.

## Discussion

**Figure 3:**
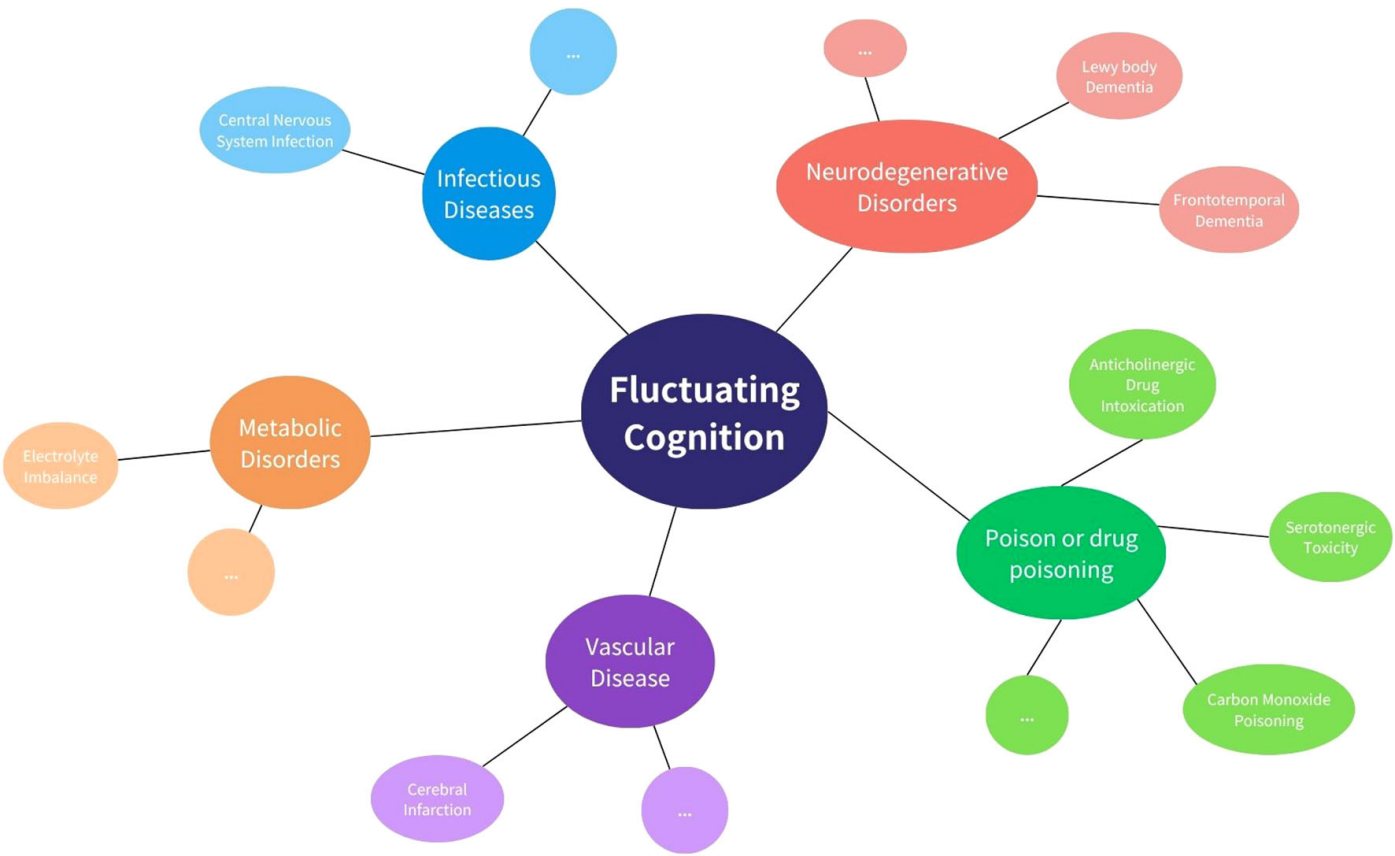
Common causes of fluctuating cognition. This diagram shows key categories of conditions that may lead to fluctuating cognition, including infectious, metabolic, vascular, neurodegenerative, and drug-related causes. Examples of each category are shown around the central concept.

While delayed encephalopathy after acute carbon monoxide poisoning (DEACMP) was ultimately diagnosed, we systematically considered and evaluated alternative etiologies that could account for the patient’s fluctuating cognition and neuropsychiatric presentation (**Figure 3**). First, vascular cognitive impairment or vascular dementia was considered, given the patient’s history of type 2 diabetes mellitus and age-related vascular risk. However, serial brain MRIs showed no evidence of progressive small vessel disease, cortical infarction, or white matter hyperintensities typical of subcortical ischemic vascular dementia. The bilateral basal ganglia lesions observed were symmetrical and characteristic of CO-related hypoxic injury rather than ischemic pathology, and they remained stable over time. Second, serotonin syndrome or serotonergic toxicity was considered due to polypharmacy. However, the patient did not exhibit hallmark features such as clonus, hyperreflexia, mydriasis, or autonomic instability. Her medication regimen was closely monitored, and serum electrolytes, liver and renal function, and ammonia levels remained within normal limits throughout hospitalization. There was no temporal correlation between medication changes and the onset of cognitive fluctuation, and the re-emergence of symptoms followed a subacute course inconsistent with serotonin toxicity. Furthermore, primary neurodegenerative disorders, such as Lewy body dementia or frontotemporal dementia, were deemed unlikely due to the absence of core features such as visual hallucinations, REM sleep behavior disorder, or prominent personality change. Cognitive testing demonstrated relatively preserved memory and language function with executive dysfunction—more consistent with toxic/metabolic encephalopathy.

Taken together, the clinical course, radiological findings, and treatment response align most consistently with DEACMP. This diagnosis is further supported by the temporal delay between CO exposure and symptom onset, the presence of characteristic MRI lesions, and partial cognitive recovery with individualized neurorehabilitation. This case presents a typical example of delayed encephalopathy due to CO poisoning, with unique individualized features worthy of further analysis.

First, the patient’s medical history reveals a two-stage pattern, with a long-standing history of depression, typical symptoms, but rapid progression of behavioral abnormalities following acute CO poisoning, suggesting the complexity and variability of neuropsychiatric symptoms in the course of CO poisoning. Acute symptoms were mild and completely resolved within a period, but over time, neuropsychiatric symptoms progressively worsened. Notably, the patient’s husband, who was exposed to the same poisoning, even experienced a coma during the acute phase but showed much milder symptoms and better recovery afterward. This phenomenon suggests that, despite being in the same environment, individual differences can significantly affect the progression of the disease.

Secondly, the clinical presentation in this case was extremely complex. Mrs. C showed significant cognitive decline following her depressive episodes, severely affecting her daily functioning, and was accompanied by abnormal behaviors and anxiety. Most notably, her disinhibited behaviors were pronounced. Although she was conscious, her responses to the environment were significantly diminished, and she exhibited emotional instability and imprecise emotional expression, often communicating in a primitive way. These symptoms intertwined with her previous depressive manifestations, making it difficult to attribute them solely to depression. Moreover, Mrs. C exhibited significant symptom fluctuations, particularly during the day, with abnormal behavior severity showing clear variation. Unlike the mood fluctuations seen in traditional psychiatric disorders such as depression or bipolar disorder, these fluctuating symptoms appeared to have an organic etiology. Observations during follow-up revealed patterns of symptom fluctuations and time distribution, suggesting a close relationship with nervous system damage. The day-to-day fluctuations in symptoms reflect the impact of organic brain injury on function, a typical hallmark of nervous system damage.

From an imaging perspective, there is no doubt that the patient’s current head MRI imaging shows residual issues. However, the classic imaging for carbon monoxide poisoning typically shows extensive demyelination of the white matter, which differs from the main manifestations in this patient. Additionally, the MRI contrast between the anterior and posterior limbs of the lateral ventricles is particularly pronounced, which cannot be explained by ordinary aging and ischemia.The effect of ischemic secondary brain damage on cortical function remains a question worthy of further exploration. Current imaging techniquesc, however, are not sufficient to reveal the detailed pathophysiological processes, further demonstrating the challenges of accurately diagnosing these cases.

From this case, we need to consider the following issues:

First of all, the case highlights the interaction between chronic mental illness and environmental toxin-induced neurological effects. Despite having the same CO exposure and initially less severe acute symptoms than her husband, the patient experienced more severe and persistent symptoms, highlighting individual differences in sensitivity to environmental toxins (3). We hypothesize that Mrs. C’s previous depression history may have made her more susceptible to brain damage from CO poisoning, with pre-existing mental illness potentially exacerbating the brain’s vulnerability to toxic effects, leading to more severe and persistent neuropsychiatric symptoms. Future research should focus on how mental illness can increase susceptibility to environmental toxins through neurobiological mechanisms and explore the prognostic differences among patients with various psychiatric conditions exposed to similar toxins. This line of research may provide new perspectives on the treatment and prevention of delayed encephalopathy due to CO poisoning and support early intervention and personalized treatment for high-risk patients.

Secondly, this case exhibits complex clinical manifestations and progression. Although the pseudo-recovery period of delayed encephalopathy is typically classified between 2 to 60 days, the patient initially displayed characteristic DEACMP symptoms one month after acute CO poisoning. After showing improvement with HBO therapy, she unfortunately relapsed a year later. This indicates that our understanding of the pseudo-recovery period may be incomplete. And her long-term symptom recurrence and atypical fluctuations post-recovery highlight the unpredictability and enduring impact of brain disease caused by carbon monoxide poisoning. This suggests that the effects of CO poisoning on brain function are more variable and prolonged than previously understood, a conclusion supported by earlier imaging findings (10).

Thirdly, DEACMP presents significant difficulties and challenges in in analyzing the etiology, as Mrs. C’s onset of re-depression was rapidly followed by cognitive decline, accompanied by disinhibited behaviors and atypical somatic symptoms. These symptoms, characterized by fluctuation and disturbances in attention and orientation, suggest multi-domain impairment. Such complexities, including severe anxiety and behaviors that cannot be fully explained by her previous depression, align with the definition of rapidly progressive dementia (RPD). The primary challenge in this case was distinguishing the recurrence of psychiatric illnesses from symptoms induced by neurological damage from CO exposure. Unlike other mental disorders, the exact neurological mechanisms of DEACMP remain elusive; nonetheless, several risk indicators have been identified, such as abnormalities in cranial CT/MRI scans (11), C-reactive protein levels (12). Rigorous investigation into the mechanisms of CO poisoning and its delayed encephalopathic manifestations, coupled with prompt and precise identification and diagnostic practices, such as the initial neurological examination (13) or laboratory test (6), are imperative for enhancing prognosis and managing the long-term consequences of this toxic exposure. A study (14) reviewed MR images obtained in 15 patients with delayed encephalopathy after acute CO intoxication and found the primary pathological characteristic of DEACMP is the reversible demyelination process in the brain’s white matter, which also be observed in this case.

Finally, current evidence for DEACMP’s treatment is still insufficient. Some studies (15, 16) suggest that neuroprotective treatments, like edaravone or memantine, contribute to the recovery of patient symptoms. Some studies (17, 18) also believe that HBO therapy may hold some significance in symptom improvement remains controversial. In this case, it did not seem to result in further improvement. The effectiveness of all these treatment methods still needs to be substantiated by more follow-up studies and evidence-based research. Mrs. C’s treatment involved an empirical delicate approach, combining antipsychotics, sedatives, antidepressants, mood stabilizers and mECT. The mechanism may involve multiple brain circuit dysfunctions, benefiting from integrative treatment like mECT. Among the pharmacological strategies employed in this case, the addition of bupropion warrants particular attention. Bupropion, a norepinephrine-dopamine reuptake inhibitor (NDRI), was introduced primarily to address persistent anxiety, affective instability, and behavioral dysregulation inadequately controlled by escitalopram monotherapy. Mechanistically, the rationale for selecting bupropion was grounded in its dual dopaminergic and noradrenergic actions, which have been hypothesized to be beneficial in disorders involving frontostriatal dysfunction, including DEACMP. Although definitive evidence regarding bupropion’s efficacy for treating motivational and executive dysfunction in DEACMP is limited, preliminary case reports and clinical trials suggest potential benefit of dopaminergic–noradrenergic modulation for related symptoms in analogous neurological disorders (19, 20). Thus, the observed clinical improvements in agitation and emotional stability in this patient, while temporally associated with the introduction of bupropion, should be interpreted cautiously, acknowledging the multifactorial nature of the treatment approach and the need for further research to clarify its specific therapeutic role in DEACMP.

Throughout the process, her condition fluctuated repeatedly. The refractoriness indicates the importance of multidisciplinary collaborative treatment. Comprehensive treatment, cognitive function management, caregiver support, and minimizing behavioral symptoms are crucial in long-term management. Patient and family education and support focus on daily care and reducing complications.

In summary, this case provides new insights into the study of DEACMP, meriting corresponding attention. Diagnosing and analyzing etiology requires detailed history-taking, comprehensive evaluations, and considering cognitive function assessment and management. For elderly individuals, depression often serves as a critical indicator of potential central nervous system pathologies which indicates early comprehensive neuropsychological evaluations are crucial. Furthermore, when feasible, it is crucial to enhance diagnostic precision through advanced imaging and biomarker assessments. This case underscores the importance of comprehensive, individualized treatment and management to alleviate symptoms, improve quality of life, and minimize long-term neurological damage. Continuous monitoring and timely adjustments based on the patient’s condition are essential for effective management.

## Data Availability

The original contributions presented in the study are included in the article/**Supplementary Material**. Further inquiries can be directed to the corresponding author.
