# Supplementary material for: Atypical progression of delayed encephalopathy post-carbon monoxide poisoning with fluctuating psychotic symptoms: a case report
Source: Front Psychiatry. 2025 Aug 1;16:1633732. doi: 10.3389/fpsyt.2025.1633732 (PMC12355180; doi:10.3389/fpsyt.2025.1633732)
Supplement: Supplementary file 1 [file SupplementaryFile1.docx]

Supplementary Material S1

S1.1 Electroencephalography

The baseline rhythm consisted predominantly of regular posterior alpha activity at 10–12 Hz with low- to medium-high amplitude (≈ 80 µV), preserved reactivity, and symmetric distribution. Scattered beta (14–30 Hz) and occasional theta waves (6–7 Hz) appeared in brief runs. Hyperventilation produced a transient theta increase that resolved spontaneously, and photic stimulation elicited no abnormalities. No epileptiform discharges or focal slowing were observed. Topographic mapping showed alpha-2 predominance. The clinical neurophysiology team interpreted the study as within normal limits.

S1.2 Near-Infrared Spectroscopy (NIRS)

According to the full NIRS report, the patient exhibited adequate cerebral blood flow fluctuations and integral values overall. However, upon task initiation, the waveform became irregular with repeated sawtooth-like patterns, increased slope, and a frontally weighted spectral center, suggesting heightened prefrontal activity. After the task, the signal largely returned to baseline, though intermittent disorganized patterns persisted. The temporal lobe signals were comparable to those of the frontal lobe, with no marked hypoperfusion.

S1.3 P300 Event-Related Potentials

P300 event-related potential testing was performed to assess cognitive processing. The main P300 component was identifiable but accompanied by substantial artefactual contamination. The waveform elicited by target stimuli demonstrated low signal-to-noise ratio and lacked clear differentiation from non-target responses. The corresponding scalp topography was atypical and lacked a clear centroparietal maximum. These findings may reflect suboptimal neural engagement during task processing.
